# Impact of climatic factors on trigeminal neuralgia and facial neuropathy in primary care during a fourteen-year time-series study

**DOI:** 10.1038/s41598-026-49760-0

**Published:** 2026-04-22

**Authors:** Juan Nicolás Cuenca-Zaldívar, Carmen Corral del Villar, Silvia García Torres, Rafael Araujo Zamora, Paula Gragera Peña, Jordi Padrós-Augé, Camilo Chávez Farías, Cristian Justribó-Manion, Eleuterio A. Sánchez-Romero, Rob Sillevis

**Affiliations:** 1 Physical Therapy Unit, Primary Health Care Center “El Abajón”, 28231 Las Rozas de Madrid, Spain; 2 Research Group in Nursing and Health Care, Puerta de Hierro Health Research Institute-Segovia de Arana (IDIPHISA), 28222 Majadahonda, Spain; 3 Interdisciplinary Research Group on Musculoskeletal Disorders, 28014 Madrid, Spain; 4 Physical Therapy Unit, Primary Health Care Center “Cerro del Aire”, 28220 Majadahonda, Spain; 5 Physical Therapy Unit, Primary Health Care Center “San Juan de la Cruz”, 28223 Majadahonda, Spain; 6 Sport, Exercise and Human Movement, Universitat de Vic – Central University of Catalonia, 08500 Vic, Spain; 7 Department of Physiotherapy, University of Vic—Central University of Catalonia, Campus UManresa, 08242 Manresa, Spain; 8 Faculty of Dentistry, Universidad Complutense de Madrid, Plaza de Ramón y Cajal s/n, 28040 Madrid, Spain; 9 Orofacial Pain Working Group, Sociedad Española de Disfunción Craneomandibular y Dolor Orofacial (SEDCYDO), 28009 Madrid, Spain; 10 Escuela Internacional de Doctorado (CEINDO), Universitat Abat Oliva CEU, CEU Universities, Bellesguard street 30, 08022 Barcelona, Spain; 11 Faculty of Economics and Social Sciences, University of Applied Sciences Osnabrück, Osnabrück, Germany; 12 Physiotherapy and Orofacial Pain Working Group, Sociedad Española de Disfunción Craneomandibular y Dolor Orofacial (SEDCYDO), 28009 Madrid, Spain; 13 Department of Rehabilitation Sciences, Florida Gulf Coast University, Fort Myers, FL 33965 USA

**Keywords:** Trigeminal neuralgia, Facial neuropathy, Biometeorology, Climatic variables, Barometric pressure, Time-series analysis, Primary care, Diseases, Health care, Neurology, Neuroscience

## Abstract

Trigeminal neuralgia and related facial neuropathic pain are severe disorders that are characterized by paroxysmal facial pain. Although meteorological factors have been associated with migraine and musculoskeletal pain, their role in trigeminal neuropathic pain remains poorly understood. Biometeorological mechanisms suggest that climatic variables may modulate symptom perception and healthcare-seeking behavior rather than disease incidence. The aim of this study was to evaluate the association between climatic variables and risk of seeking primary care for trigeminal neuralgia and facial neuropathy over a 14-year period in a Mediterranean population. This retrospective cohort study was conducted using electronic health records from three primary care centers in Madrid (Spain) between 2010 and 2023. All consultations coded as trigeminal neuralgia (ICPC-2 N03) or facial neuropathy (ICPC-2 N89.02) were included in the study. Individual consultation events were linked to meteorological data from the Spanish State Meteorological Agency. A piecewise additive exponential event-based model fitted with a negative binomial distribution was applied to estimate hazard ratios (HR), allowing flexible modeling of non-linear climatic effects and long-term temporal trends in a low-event-density, time-to-event context. A total of 246 consultations were identified (mean age 66.6 ± 14.6 years; 66.7% female). Increased hours of sunshine were independently associated with a higher risk of seeking medical attention for trigeminal neuralgia (HR = 1.081 per additional hour; 95% CI, 1.016–1.150). A significant non-linear association with ambient temperature was observed, with higher consultation risk at temperatures below approximately 10 °C and stabilization thereafter. The long-term temporal trend remains largely stable until there is a significant increase in consultation risk during the post-COVID-19 period (2021–2023). In this population-based primary care study, specific climatic factors, particularly sunlight exposure and low ambient temperature, were associated with the risk of seeking medical attention for trigeminal neuralgia and facial neuropathic pain. These findings suggest that climatic variables may act as modulators of healthcare utilization rather than primary drivers of disease incidence. Intrinsic neuropathological mechanisms remain central to trigeminal neuralgia pathophysiology, whereas environmental factors may influence symptom perception and care-seeking behavior in susceptible individuals.

**Supplementary Information:**

The online version contains supplementary material available at 10.1038/s41598-026-49760-0.

## Introduction

Humans continuously interact with their surrounding environment, and climatic variability has long been recognized as a determinant of health and disease patterns across populations^1^. Biometeorology, a scientific discipline that explores the interaction between atmospheric conditions and living organisms, provides an essential framework for understanding how meteorological fluctuations modulate physiological and pathological processes^2^. Within this field, *meteoropathy* refers to the onset or exacerbation of symptoms triggered by changes in weather conditions^3^. It is estimated that approximately one-third of the population exhibits some degree of weather sensitivity^4^, and up to three-quarters of patients with chronic pain report symptom fluctuations linked to meteorological changes^5^.

Environmental and atmospheric factors can modulate pain via distinct biological pathways. Several primary headache disorders, such as cluster headache and, to a lesser extent, migraine, exhibit clear seasonal and circadian patterns^6,7^. These conditions are influenced by cyclic variations in daylight exposure, temperature, and atmospheric pressure, which interact with hypothalamic structures that regulate the sleep–wake cycle, melatonin secretion, and vascular tone^6,7^. This rhythmic modulation contributes to the characteristic seasonal clustering observed in cluster headache and the periodicity described in some migraine phenotypes, consistent with recent app-based analyses showing cyclical peaks of headache occurrence during transitional weather periods^8^.

Mechanisms that are not directly linked to biological rhythms may also influence pain through acute environmental fluctuations^9^. Sudden decreases in barometric pressure^10^, variations in ambient oxygen or carbon dioxide levels, and changes in humidity or wind speed can alter cerebral perfusion, vascular resistance, and tissue oxygenation, thereby modulating neuronal excitability and pain perception^9,11^. These factors can influence neuroendocrine and autonomic regulation^12,13^, and alter mood, vascular tone, and pain perception^11,13^. Experimental evidence suggests that abrupt changes in atmospheric pressure can activate brainstem nuclei involved in vestibular and trigeminal sensory processing^14^, supporting a mechanistic basis for climate-related pain modulation.

​Trigeminal neuralgia (TN) is a chronic neuropathic pain disorder characterized by recurrent unilateral paroxysmal attacks along one or more branches of the trigeminal nerve^15^ that can sometimes be spontaneous, but is mostly precipitated by innocuous stimuli within the affected trigeminal distribution^16^. It is among the most severe facial pain conditions, with a prevalence estimated between 0.03% and 0.3%, showing a female-to-male ratio of approximately 3:1, and most frequently affecting adults aged 37–67 years^17^. These data were derived from population-based studies summarized in a systematic review of over 13,000 adults, confirming a higher involvement of the maxillary and mandibular branches of the trigeminal nerve^16,17^.

The pathophysiology of TN involves peripheral or central sensitization of trigeminal afferents resulting from mechanisms such as focal demyelination, neuroinflammatory changes, or abnormal ion channel activity within the trigeminal root entry zone. While neurovascular compression of the trigeminal nerve is characteristic of the classical form, it is not present in all patients and does not explain the diverse clinical presentation of the disorder^18^. As these mechanisms are highly sensitive to fluctuations in temperature, pressure, and humidity, meteorological variability may act as a precipitating or exacerbating factor for TN episodes^13^.​

Although these pathophysiological mechanisms suggest that trigeminal pain may be sensitive to environmental modulation, empirical data directly examining this relationship are limited. Existing studies have primarily relied on small clinical samples or self-reported triggers, with heterogeneous methodologies and limited integration of objective meteorological data. Consequently, the true impact of climatic fluctuations on the onset or exacerbation of TN remains unclear.

Observational evidence exploring the influence of climatic or atmospheric changes on TN remains scarce. In a hospital-based cross-sectional study, Ayele et al.^19^ collected demographic and clinical data from 61 patients with TN and noted that a subset reported seasonal variation in pain, although no meteorological variables were objectively analyzed. This finding supports the notion that environmental factors may modulate the symptom patterns in some patients.

Similarly, Koh et al.^20^ conducted a structured interview study of 60 patients with TN to identify atypical triggers, including food and weather-related factors. Approximately 20% of the participants reported meteorological triggers, most frequently cold air, strong wind, or sudden temperature changes, which the authors hypothesized could activate A-delta sensory afferents involved in mechanothermal nociception. The study’s cross-sectional and self-reported design, absence of objective weather data, and small sample size limit causal inference but highlight a plausible peripheral mechanism for environmental pain modulation.

Extending this concept, Ben-Ari et al.^21^ performed a meta-analysis of studies describing facial and TN induced by atmospheric pressure changes (e.g., during aviation, diving, or hyperbaric exposure). Their pooled results demonstrated an association between barometric fluctuations and cranial nerve dysfunction, providing indirect support for pressure-related modulation of trigeminal pain. However, the authors emphasized the heterogeneity and limited quality of the available studies, most of which were case reports or small observational series, and cautioned that experimental contexts involving abrupt, extreme pressure changes differ substantially from natural meteorological variations.

In fact, the perception of somatosensory alterations in the facial area evoked by cold and warm weather has been proposed in screening protocols for orofacial pain conditions, as it is known that primary nociceptive neurons can be stimulated by α-δ and C fibers that respond to thermal stimuli^22^.

Collectively, these studies indicate that while a proportion of TN patients report weather-related symptom exacerbation and there is some evidence linking barometric variation to cranial nerve irritability, the existing literature remains methodologically limited, predominantly descriptive, and calls for prospective studies that incorporate objective environmental data and standardized pain outcomes.

Understanding the potential relationship between climate and TN is clinically relevant, as it may contribute to identifying high-risk periods for symptom exacerbation and improving the timing of preventive interventions^20,21^. Moreover, integrating climatic data into predictive health models could help anticipate peaks in facial pain consultations, aiding healthcare resource planning and patient counseling^20,21^. However, despite biological plausibility and emerging observational evidence, no long-term population-based studies have systematically evaluated how objective climatic variables relate to the healthcare demand for TN, leaving the epidemiological impact of environmental fluctuations largely unquantified.

Therefore, this study aimed to analyze the association between climatic variables (temperature, precipitation, wind direction and speed, sunshine hours, and barometric pressure) and the weekly number of primary care consultations for trigeminal neuralgia from 2010 to 2023. The secondary objectives were to evaluate the influence of age and sex on these associations, and to describe the temporal trends in consultation rates across the 14-year observation period.

## Methods

### Data source and study population

A retrospective cohort study was conducted using data extracted from the electronic health records of patients from three primary care centers in Madrid, Spain: *“El Abajón”* (Las Rozas), *“Cerro del Aire”* (Majadahonda), and *“San Juan de la Cruz”* (Pozuelo de Alarcón).

The study period extended from January 1, 2010, to December 31, 2023, and included all patients aged 18 years or older who consulted for trigeminal neuralgia or facial neuropathy during this period. Diagnoses were identified using the *International Classification of Primary Care*,* Second Edition (ICPC-2)*, specifically codes N03 (Trigeminal neuralgia) and N89 (locally subclassified as N89.02 in the database).

Facial neuropathy was operationally defined according to the International Classification of Primary Care and Second Edition (ICPC-2). Specifically, the code N89.02 was used to identify non-dental painful facial conditions recorded in primary care that do not strictly fulfill the classical diagnostic criteria for trigeminal neuralgia. This category may include idiopathic, post-inflammatory, or otherwise unspecified facial neuropathic pain presentations, as is routinely coded in primary care settings.

In routine primary care practice, the ICPC-2 code N89.02 is frequently used as a pragmatic diagnostic category when patients present with neuropathic facial pain symptoms that overlap clinically with trigeminal neuralgia but lack sufficient information at first contact to allow strict nosological classification. This approach reflects real-world diagnostic behavior in primary care, where early presentations, atypical distributions, or incomplete diagnostic workup often preclude immediate differentiation between classical trigeminal neuralgia and other facial neuropathic pain syndromes.

For the purposes of the present analysis, consultations coded as trigeminal neuralgia (N03) and facial neuropathy (N89.02) were analyzed together, as both represent trigeminally mediated neuropathic facial pain conditions managed in primary care. This strategy was adopted to reflect healthcare demand rather than strict etiological classification and to maximize the capture of clinically relevant trigeminal pain presentations in the early or undifferentiated stages of the disease.

Sociodemographic variables (age and sex) were retrieved from electronic medical records and the corresponding consultation date. Meteorological data were obtained from the *Spanish State Meteorological Agency* (AEMET) station in Pozuelo de Alarcón (ID3194Y; latitude 40º26′54″N, longitude 3º48′48″W), located within 15 km of all the participating centers. The total catchment area covers approximately 140 km².

This study was approved by the *Research Ethics Committee of Puerta de Hierro Majadahonda Hospital* (approval code: PI 70/24, Act 06/2024). All the procedures adhered to the *Declaration of Helsinki* and ensured patient anonymity and confidentiality. The manuscript follows the *Strengthening the Reporting of Observational Studies in Epidemiology (STROBE)* statement^23^.

### Climatic variables

Meteorological data corresponding to the same geographical region and timeframe were obtained from AEMET. The variables collected included mean, maximum, and minimum temperatures (°C); precipitation (L/m²); wind direction (°); mean and gust wind speed (m/s); maximum and minimum barometric pressure (hPa); and sunshine hours (h/day).

### Follow-up process for consultations

All primary care consultations for trigeminal neuralgia or facial neuropathy recorded between 2010 and 2023 were compiled to describe the temporal patterns of consultations over the study period; inferential analyses were conducted using an event-based time-to-consultation approach. This longitudinal dataset enables the assessment of temporal fluctuations, seasonality, and recurrence patterns in healthcare utilization.

**Fig. 1:**
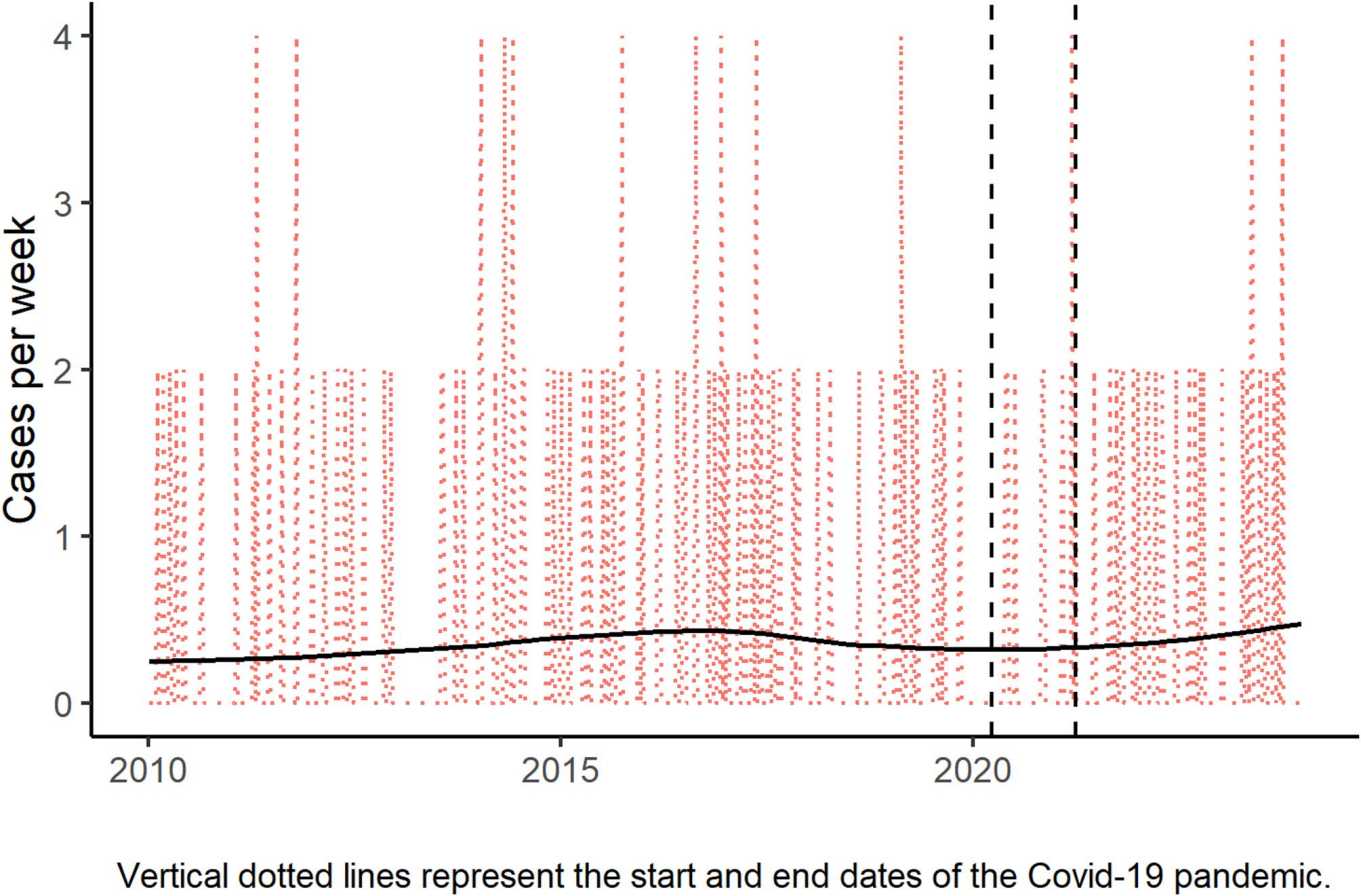
Temporal distribution of consultations for trigeminal neuralgia and facial neuropathy (2010–2023). Dots represent the number of consultations aggregated per week for visualization of long-term temporal patterns, and the solid line indicates the smoothed trend. Weekly aggregation is shown solely for descriptive graphical representation; statistical analyses were performed using individual consultation events within the piecewise additive exponential modeling framework. Vertical dashed lines indicate the approximate start (March 2020) and end (2021) of the COVID-19 pandemic period.

For descriptive visualization purposes, consultation counts were also aggregated weekly in Fig. 1 to illustrate long-term temporal patterns; however, all inferential analyses were conducted using individual consultation events in the event-based modeling framework.

By linking demographic and meteorological variables, this study evaluated the environmental influences on healthcare utilization related to trigeminal and facial neuropathic pain in real-world primary care settings.

### Sample size

According to Burmeister et al.^24^, at least 240 observations are required to ensure adequate power in multiple regression models with several predictors, which are mainly applied to cross-sectional analyses.

### Study procedures

Patient data, including age, sex, and ICPC-2 diagnostic codes, were anonymized and extracted from the electronic records. These variables were systematically matched with the meteorological data obtained from the AEMET for the same period.

The climatic dataset included the mean, maximum, and minimum temperatures (°C), precipitation (L/m²), wind direction (°), mean and gust wind speeds (m/s), sunshine hours (h/day), and maximum and minimum barometric pressures (hPa)^9,10,13,21^.

### Outcome measures

The primary outcome was the occurrence of primary care consultation for trigeminal neuralgia or facial neuropathic pain (healthcare-seeking behavior), modeled as a time-to-event process, as identified by ICPC-2 diagnostic codes. Secondary analyses evaluated the influence of sex, age, and meteorological variables on temporal consultation trends, and explored possible seasonal or environmental triggers for facial pain exacerbations. The diagnostic criteria were consistently applied over the 14-year period, and all meteorological variables followed the AEMET operational standards to ensure homogeneity and reproducibility.

### Statistical analysis

Statistical analyses were performed using R version 4.1.3 (R Foundation for Statistical Computing, Vienna, Austria). The significance level was set at *P* < 0.05.

Quantitative variables are described as mean±standard deviation, and qualitative variables are described as absolute and relative values (%).

The primary outcome of this study was the risk of seeking medical attention for trigeminal neuralgia or facial neuropathic pain, which was used as a proxy for symptom exacerbation. Given the low frequency and sparse temporal distribution of consultations, with a high proportion of zero counts across weekly observations, conventional time-series approaches based on aggregated counts and normality assumptions (e.g., ETSX or ARIMAX models) were deemed inappropriate. Therefore, an event-based modeling strategy was adopted. To assess the association of this risk with weather conditions, age, and sex, a Piecewise Additive Exponential Model (PAM) with shrinkage penalty was applied, allowing the calculation of the Hazard Ratio (HR) associated with seeking medical attention. Rather than aggregating consultations into weekly counts, each consultation was treated as an individual time-to-event observation, allowing direct modeling of event occurrence without imposing artificial temporal averaging. This approach transforms survival data into a format that allows time to be treated as a continuous variable, optimizing the statistical power in a low-event-density context^25^. Given the high variability and overdispersion observed in the frequency of seeking medical attention, the model was fitted using a Negative Binomial (NB) distribution, which provides a more robust estimation of standard errors than the Poisson distribution^26,27^. The complexity of the predictor variables was assessed using the Effective Degrees of Freedom (EDF), where a value of 1-1.5 was considered indicative of linear behavior, values greater than 1.5 indicated non-linear relationships modeled by splines, and values < 0.5 implied the exclusion of the variable, as its effect had been penalized until it was null^26,28^.

The dataset included all recorded consultations per patient, allowing individuals to contribute to more than one consultation during the study period. To ensure that repeated visits did not introduce dependency bias, residual autocorrelation was formally assessed using the Ljung–Box test^29^. For non-linear variables, concurvity was evaluated, eliminating those with values greater than 0.8, and the sufficiency of the number of basic functions was confirmed using the K index (*p* > 0.05). For linear variables, the Variance Inflation Factor (VIF) was evaluated, and those with values greater than 5.

## Results

A total of 246 consultations for trigeminal neuralgia or facial neuropathy were identified during the 14-year observation period, with a mean age of 66.64 ± 14.57 years and a predominance of female patients (66.67%) (Table 1).

**Table 1:** Descriptive characteristics of consultations and climatic conditions included in the analysis (2010–2023).

*n*	Overall	2010	2011	2012	2013	2014	2015	2016	2017	2018	2019	2020	2021	2022	2023
	246	12	18	12	8	20	20	24	26	10	22	10	20	20	24
*Socio-demographic characteristics*															
Age	66.64 ± 14.57	60.67 ± 15.79	67.11 ± 17.61	72.17 ± 8.54	72.25 ± 14.88	71.20 ± 10.76	63.90 ± 12.04	68.08 ± 14.73	70.54 ± 9.79	67.00 ± 9.48	63.45 ± 14.58	71.40 ± 15.24	66.30 ± 13.47	64.90 ± 14.74	60.00 ± 21.61
Gender (Female)	164 (66.67)	10 (83.33)	12 (66.67)	8 (66.67)	4 (50)	16 (80)	14 (70)	14 (58.33)	14 (53.85)	10 (100)	20 (90.91)	6 (60)	10 (50)	12 (60)	14 (58.33)
Gender (Male)	82 (33.33)	2 (16.67)	6 (33.33)	4 (33.33)	4 (50)	4 (20)	6 (30)	10 (41.67)	12 (46.15)	0 (0)	2 (9.09)	4 (40)	10 (50)	8 (40)	10 (41.67)
*Atmospheric conditions*															
Average temperature (degrees Celsius)	14.99 ± 7.66	14.74 ± 8.41	15.53 ± 7.36	14.13 ± 7.91	13.77 ± 7.73	14.91 ± 6.74	15.28 ± 7.74	14.73 ± 7.61	15.28 ± 7.87	14.57 ± 7.80	15.12 ± 7.38	15.07 ± 7.37	14.95 ± 7.29	16.14 ± 7.93	15.69 ± 7.77
Average rainfall (l/m²)	1.43 ± 4.84	2.02 ± 5.44	1.26 ± 3.66	1.09 ± 4.40	1.41 ± 4.48	1.48 ± 4.46	0.92 ± 3.21	1.84 ± 4.97	0.85 ± 3.23	1.94 ± 5.46	1.26 ± 4.91	1.40 ± 4.39	1.28 ± 4.74	1.48 ± 4.22	1.79 ± 8.18
Average wind speed (mtrs/sec)	2.87 ± 1.77	2.71 ± 1.77	2.54 ± 1.61	2.91 ± 1.84	2.90 ± 1.90	3.09 ± 1.88	2.68 ± 1.72	2.73 ± 1.92	2.55 ± 1.75	3.01 ± 1.71	3.29 ± 1.91	2.96 ± 1.75	2.94 ± 1.60	2.83 ± 1.54	3.01 ± 1.73
Wind gusts (m/s)	10.03 ± 3.64	10.09 ± 3.55	9.67 ± 3.32	10.26 ± 3.60	10.52 ± 3.57	10.60 ± 3.59	9.72 ± 3.74	9.89 ± 3.67	9.94 ± 3.67	10.10 ± 3.49	10.58 ± 4.07	9.75 ± 3.79	9.90 ± 3.47	9.92 ± 3.46	9.56 ± 3.82
Sunshine hours	8.20 ± 3.98	7.79 ± 4.26	8.30 ± 4.05	8.47 ± 3.92	8.16 ± 4.02	8.10 ± 3.97	8.31 ± 3.77	7.97 ± 4.17	8.75 ± 3.59	8.05 ± 3.95	8.82 ± 3.78	7.89 ± 4.20	7.86 ± 3.93	7.93 ± 4.26	8.46 ± 3.57
Diurnal temperature range (degrees Celsius)	13.74 ± 4.93	10.94 ± 3.82	12.90 ± 4.51	14.62 ± 5.10	13.68 ± 4.85	13.28 ± 4.80	13.99 ± 4.79	13.79 ± 5.25	15.47 ± 4.60	13.29 ± 4.73	14.47 ± 5.15	13.38 ± 5.08	13.95 ± 4.92	13.90 ± 4.93	14.64 ± 4.97
Day-to-day temperature change (degrees Celsius)	0.00 ± 1.97	0.01 ± 2.05	-0.01 ± 1.77	-0.01 ± 2.03	0.01 ± 2.04	0.01 ± 1.81	0.00 ± 2.08	-0.02 ± 1.85	0.02 ± 2.15	0.00 ± 1.98	0.00 ± 2.05	-0.01 ± 2.01	0.02 ± 1.99	0.00 ± 1.91	-0.01 ± 1.87
Average barometric pressure (hPa)	4.38 ± 2.30	4.95 ± 2.85	4.11 ± 1.91	4.21 ± 1.81	4.60 ± 2.65	4.43 ± 2.76	4.36 ± 2.22	4.52 ± 2.29	4.44 ± 2.46	4.56 ± 2.48	4.57 ± 2.45	4.27 ± 2.11	4.11 ± 1.98	4.15 ± 1.72	4.08 ± 2.04
Day-to-day barometric pressure change (hPa)	0.00 ± 2.71	-0.01 ± 3.34	0.01 ± 2.39	0.00 ± 2.24	0.01 ± 3.03	0.00 ± 2.95	0.01 ± 2.67	-0.01 ± 2.81	0.02 ± 2.79	-0.02 ± 2.91	0.00 ± 2.89	0.00 ± 2.62	0.00 ± 2.36	0.01 ± 2.26	0.00 ± 2.46
Wind direction (cosine)	0.89 ± 0.22	0.93 ± 0.05	0.94 ± 0.05	0.94 ± 0.05	0.94 ± 0.05	0.94 ± 0.04	0.94 ± 0.05	0.94 ± 0.05	0.92 ± 0.14	0.89 ± 0.23	0.86 ± 0.27	0.83 ± 0.33	0.78 ± 0.38	0.82 ± 0.35	0.81 ± 0.33
Wind direction (sine)	0.34 ± 0.20	0.32 ± 0.15	0.32 ± 0.14	0.32 ± 0.15	0.31 ± 0.16	0.32 ± 0.14	0.31 ± 0.15	0.31 ± 0.15	0.31 ± 0.17	0.35 ± 0.20	0.37 ± 0.21	0.37 ± 0.25	0.41 ± 0.27	0.38 ± 0.26	0.42 ± 0.24

Data are expressed as mean ± standard deviation or as absolute and relative values (%). These variables describe the demographic characteristics of consultations and the meteorological context of the study period. The piecewise additive exponential model was applied in subsequent inferential analyses using individual consultation events rather than these aggregated descriptive summaries.

Over time, a generally stable trend was observed in the number of trigeminal-neuralgia consultations, with a modest decline in 2015 and a return to baseline levels in subsequent years (Fig. 1).

After evaluation of the initial model, age, average rainfall, average wind speed, diurnal temperature range, average barometric pressure, and wind direction were excluded because of penalization or lack of contribution to the model fit (Supplementary Table 1). The final model showed no concurvity among smoothed terms, adequate basis dimensions, absence of multicollinearity among linear predictors, and no residual autocorrelation. Overdispersion was observed, justifying the use of a negative binomial distribution (Supplementary Table 2).

The final model indicated that hours of sunlight were a significant linear predictor of the risk of seeking medical attention for trigeminal neuralgia, with an increase of 8.1% for each additional hour of sunlight [HR = 1.081 (95% CI: 1.016–1.150)]. Regarding nonlinear effects, both the temporal trend (EDF = 4.343 (9), *p* < 0.001) and mean temperature (EDF = 1.8 (2), *p* < 0.001) were significantly associated with the risk of seeking medical attention (Table 2).

**Table 2:** Trigeminal neuralgia final model summary.

Linear terms		
	Hazard ratio (95% CI)	Z-score (^a^p value)
(Intercept)	(baseline hazard, not directly interpretable)	Z = −20.695, *p*** <** **0.001**
Sunshine hours	1.081 (1.016, 1.15)	Z = 2.446, *p* = **0.014**
Wind gusts (m/s)	1.044 (0.991, 1.1)	Z = 1.636, *p* = 0.102
Gender (Male)	0.79 (0.538, 1.16)	Z = −1.201, *p* = 0.23

Smoothed terms		
	EDF (df_ref_)	X^2^ (^a^p value)
Time trend	4.343 (9)	X^2^(9) = 13.152, *p* **<** **0.001**
Average temperature (degrees Celsius)	1.8 (2)	X^2^(2) = 7.843, *p* **<** **0.001**

95% CI: 95% confidence interval; EDF: Effective degrees of freedom, dfref: Reference degrees of freedom.

^a^significant if p < 0.05 (shown in bold).

**Fig. 2:**
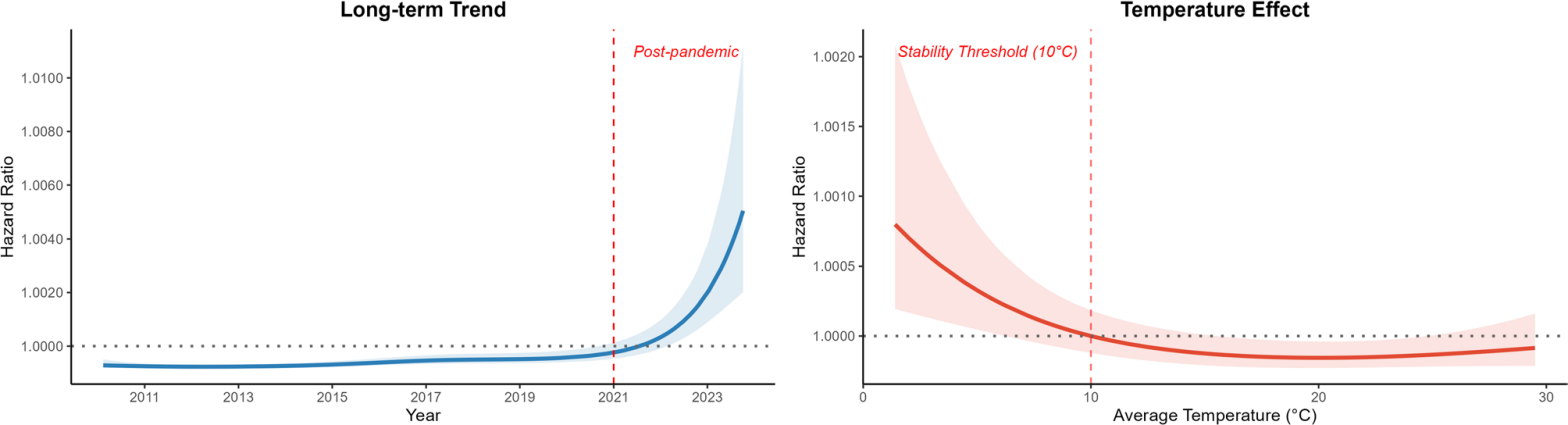
Partial effects of time and temperature on the risk of seeking primary care for trigeminal neuralgia and facial neuropathy. Left panel: smoothed long-term temporal effect expressed as hazard ratio (HR), highlighting the post-pandemic period. Right panel: non-linear association between average temperature and consultation risk, showing a higher risk at lower temperatures and stabilization beyond approximately 10 °C. The shaded areas represent 95% confidence intervals.

The long-term trend showed a stable risk of seeking medical attention for most of the follow-up period, followed by a sudden and significant increase in the final segment (2021–2023), coinciding with the period after the COVID-19 pandemic. On the other hand, an inverse relationship between temperature and the risk of seeking medical attention is evident; the hazard ratio decreases progressively as the temperature increases until reaching the 10 °C threshold, after which the risk stabilizes (Fig. 2).

## Discussion

### Principal findings

This 14-year time-series analysis evaluated the association between climatic variables and the risk of seeking primary care for trigeminal neuralgia and facial neuropathic pain using a piecewise additive modeling approach specifically designed to address low event density and non-linear temporal dynamics. Unlike conventional time-series forecasting models, this analytical strategy allows the identification of selective and clinically interpretable associations between specific environmental factors and consultation risk.

Increased hours of sunshine was independently associated with a higher risk of seeking medical attention for trigeminal neuralgia. This finding suggests that higher light exposure or behavioral patterns linked to clear days, such as increased outdoor activity, sensory stimulation, or changes in daily routines, may act as facilitating factors for susceptible individuals. Alternatively, greater sunlight exposure may reduce environmental or logistical barriers to healthcare access, thereby increasing the likelihood of consultation without necessarily reflecting a higher underlying disease incidence^30^.

A non-linear association was observed between ambient temperature and consultation risk. The highest risk of seeking medical attention occurred at temperatures below approximately 10 °C, followed by a progressive stabilization as temperatures increased. This pattern supports the hypothesis that cold exposure may contribute to trigeminal pain exacerbations, whereas temperate conditions may attenuate this effect, consistent with previous evidence linking cold-related stimuli to craniofacial pain modulation^31,32^.

Additionally, a marked increase in consultation risk was observed during the post–COVID-19 period. This temporal shift may reflect delayed healthcare-seeking behavior during the pandemic, increased awareness and reporting of neuropathic symptoms, or potential neuroinflammatory and sensory sequelae associated with post-acute COVID-19 manifestations affecting the cranial nerves^32,33^. Importantly, this finding highlights a change in healthcare utilization patterns rather than a direct causal effect of infection, and should be interpreted within the broader context of pandemic-related healthcare disruption.

Overall, these findings indicate that, while intrinsic neuropathological mechanisms remain central to trigeminal neuralgia, certain climatic variables, particularly sunlight exposure and low ambient temperature, may act as modulators of symptom perception and healthcare-seeking behavior in a subset of patients. Consistent with prior biometeorological research, climatic influences appear to operate indirectly and heterogeneously across individuals^9^, exerting a measurable effect on consultation risk without constituting the primary drivers of disease occurrence at the population level.

### Comparison with previous studies

Previous research has consistently highlighted the potential influence of meteorological factors on chronic pain conditions, particularly migraines, fibromyalgia, and musculoskeletal disorders^4,5,8,9,11–14,21^. In these conditions, associations between pain exacerbations and variables such as temperature, barometric pressure, humidity, and sunlight exposure have been reported, although the magnitude and reproducibility of these effects remain heterogeneous.

In contrast, evidence specifically addressing cranial neuropathic pain and trigeminal neuralgia is comparatively scarce and inconclusive. Most available studies rely on small clinical samples, patient-reported trigger descriptions, or indirect mechanistic hypotheses rather than long-term population-based data^19–21^. Experimental and clinical investigations suggest that abrupt changes in barometric pressure and ambient temperature may influence trigeminal excitability and craniofacial sensory processing. For instance, reductions in atmospheric pressure have been shown to activate the vestibular and trigeminal nuclei in animal models^10^, and several biometeorological mechanisms related to temperature, humidity, and pressure have been proposed to modulate pain physiology^9,11^. Moreover, meta-analytic evidence has linked extreme or rapid pressure fluctuations to facial and trigeminal neuropathies^21^.

Similarly, cold exposure and thermal or wind-related stimuli have been proposed as potential facilitators of craniofacial pain by lowering pain thresholds through A-δ- and C-fiber-mediated mechanothermal nociception, as described in the standardized somatosensory assessment frameworks for orofacial pain^22^. These mechanisms provide biological plausibility for the effect of low ambient temperature on trigeminal pain expression, which is consistent with the increased consultation risk observed at temperatures below approximately 10 °C in the present analysis.

Nevertheless, long-term observational studies examining weather–pain relationships have frequently reported weak, inconsistent, or highly variable associations, emphasizing substantial inter-individual heterogeneity^11,12,34,35^. Large-scale analyses and methodological reviews have repeatedly shown that climatic influences on pain tend to be modest at the population level and are difficult to reproduce across settings, even in conditions traditionally considered weather-sensitive.

The present findings are consistent with this body of literature, suggesting that climatic factors may act as modulators rather than primary drivers of trigeminal neuralgia–related healthcare utilization. While specific associations were identified with sunlight exposure and low ambient temperature, these effects were selective and non-linear, supporting the notion that environmental influences operate indirectly and may interact with behavioral or contextual factors rather than reflecting direct disease causation.

A relevant point of comparison emerges when contrasting these results with our previously published 14-year population-based study on chronic musculoskeletal pain in Spanish primary care^36^. In that cohort, lower temperatures were associated with increased consultations for shoulder and arm pain, indicating stronger and more consistent population-level sensitivity to climatic conditions. In contrast, the current study demonstrated more limited and condition-specific climatic associations between trigeminal neuralgia and facial neuropathic pain.

This divergence likely reflects fundamental differences in the underlying pathophysiology and healthcare utilization patterns. Musculoskeletal pain is closely linked to peripheral tissue sensitivity, inflammatory processes, and biomechanical loading, which may be directly influenced by ambient temperature. In contrast, trigeminal neuralgia is predominantly driven by intrinsic neuropathological mechanisms, such as neurovascular compression, focal demyelination, and aberrant neural excitability^15,18,37–39^, which may attenuate the impact of moderate climatic variation at the population level.

Importantly, both studies were conducted over a comparable 14-year period (2010–2023), using routinely collected primary care data from the Madrid region. Meteorological variables in both analyses were obtained from official AEMET stations within the metropolitan area, which share a continental Mediterranean climate characterized by similar temperature ranges, seasonal patterns, and interannual variability. Although different stations were used to optimize the proximity to healthcare catchment areas, the overall climatic exposure was broadly comparable. Therefore, the contrasting associations observed between musculoskeletal pain and trigeminal neuropathic pain are unlikely to be explained by differences in geography, timeframe, or weather conditions, and instead underscore condition-specific pathophysiological and healthcare-seeking dynamics.

### Mechanistic considerations

Trigeminal neuralgia is primarily attributed to focal demyelination or compression of the trigeminal root entry zone, leading to ephaptic transmission and marked hyperexcitability of the trigeminal afferent fibers^15,18,37–39^. These intrinsic neuropathological mechanisms are dominant drivers of symptom generation and recurrence. Although environmental variables, such as temperature, barometric pressure, or light exposure, may modulate peripheral or central sensory processing, their contribution is expected to be secondary when compared with well-established neurophysiological processes^9,11,12^.

Facial neuropathic pain syndromes grouped under ICPC-2 code N89.02 may differ from classical trigeminal neuralgia, potentially involving post-inflammatory nerve injury, peripheral sensitization, or mixed nociceptive–neuropathic mechanisms. Nevertheless, these conditions converge on shared trigeminal pathways and central sensory processing circuits, supporting their joint analysis when the outcome of interest is healthcare utilization related to trigeminal pain, rather than precise etiological differentiation^16,22^.

Experimental and translational studies have demonstrated that decreases in barometric pressure can activate neurons within the vestibular and trigeminal nuclei^10^ and that fluctuations in temperature, humidity, and atmospheric conditions may influence autonomic tone, vascular regulation, and nociceptive processing^4,13,14^. These mechanisms provide biological plausibility for environmental modulation of trigeminal excitability. However, such effects do not necessarily translate into clinically meaningful fluctuations in population-level consultation rates, particularly for large administrative datasets.

Several factors may explain why environment-driven neurophysiological modulation does not consistently generate strong consultation signals at the population level. First, patients with established trigeminal neuralgia frequently develop behavioral adaptations, including avoidance of known triggers such as cold exposure or outdoor activity during adverse conditions, which may attenuate symptom exacerbations before medical contact occurs^20^. Second, the threshold for seeking care is strongly influenced by individual pain tolerance, coping strategies, prior diagnostic pathways, and ongoing pharmacological management, which introduces substantial variability between symptom onset and consultation timing. Third, the latency between paroxysmal pain episodes and primary care consultation may obscure short-term meteorological effects, particularly when analyses rely on healthcare utilization rather than real-time symptom reporting.

In addition, the Mediterranean climate characteristic of the study region is marked by relatively moderate thermal ranges and limited extremes, which may further constrain the detectability of large climate-related shifts in consultation behavior at the population level. Even when biological sensitivity exists at the individual level, moderate climatic variability may not be sufficient to generate pronounced aggregate effects.

Chronic orofacial pain also involves complex interactions between the somatosensory cortical networks, limbic circuitry, and descending inhibitory pathways, which are modulated by genetic, psychological, and environmental factors^15,16,40^. Importantly, sensory phenotypes differ across pain conditions, and musculoskeletal pain often manifests predominantly as gain-of-function phenomena such as hyperalgesia or hyperesthesia, whereas neuropathic pain syndromes frequently combine sensory loss (hypoesthesia) with positive sensory symptoms, including allodynia and paroxysmal hyperalgesia^22^. These differences may partially explain why climatic factors appear to exert stronger population-level effects on musculoskeletal pain than on trigeminal neuropathic conditions^12,34,36^.

Finally, heterogeneity across trigeminal neuropathic phenotypes, including ophthalmic-predominant presentations with distinct sensory profiles, further underscores the complexity of the underlying mechanisms^41^. Such interindividual variability in meteorosensitivity and neural vulnerability may dilute subtle climatic effects when data are aggregated across heterogeneous patient populations.

### Clinical and public health implications

From a clinical perspective, the identification of specific climatic associations in this study, particularly hours of sunlight, lower ambient temperatures, and the post–COVID-19 period—suggests that environmental factors may act as contextual modulators of healthcare-seeking behavior in patients with trigeminal neuralgia and related facial neuropathic pain rather than as primary determinants of disease incidence. Importantly, the presence of statistically significant associations at the population level did not imply uniform meteorosensitivity across patients.

Therefore, clinicians should remain attentive to individual variability in weather sensitivity. Previous literature indicates that certain subgroups, including older adults and patients with comorbid affective, sleep, or somatic symptom disorders, may be more likely to perceive climatic change as a pain-aggravating factor^1,3,5^. Incorporating biometeorological awareness into clinical interviews may be useful, particularly for patients who report reproducible symptom exacerbations linked to cold exposure, seasonal transitions, or environmental conditions.

The observed increase in consultation risk at temperatures below 10 °C supports practical counseling strategies focused on thermal protection. Advising patients to maintain facial warmth, minimizing exposure to cold air or wind, and using protective clothing during colder months may help reduce symptom provocation in susceptible individuals, even if such measures do not alter the underlying neuropathological processes. Similarly, the association between increased hours of sunlight and consultation risk may reflect behavioral factors, such as increased outdoor activity or reduced barriers to accessing healthcare, rather than a direct photic trigger, underscoring the importance of interpreting climatic associations within a broader behavioral and healthcare utilization framework^30^.

A significant increase in consultation risk during the post–COVID-19 period has potential implications for clinical vigilance and service planning. Emerging evidence suggests that neuropathic pain may occur as part of post-acute COVID-19 manifestations, including isolated trigeminal neuralgia^32,33^. While causal inference could not be established in the present study, clinicians should be aware of the potential overlap between post-viral neurological sequelae and trigeminal pain syndromes, particularly in patients presenting with new-onset or atypical symptom patterns after the pandemic period.

Despite these observations, the present findings reinforce that the cornerstone of clinical management of trigeminal neuralgia and facial neuropathic pain should remain focused on intrinsic neuropathological mechanisms. Neurovascular compression, focal demyelination, aberrant ion-channel activity, and central sensitization processes continue to be the primary drivers of symptom severity and recurrence and should guide diagnostic evaluation and therapeutic decision-making^15,18,37–39^. Climatic and environmental factors are best considered secondary modulators that may influence symptom perception or consultation timing in selected individuals rather than as primary therapeutic targets at the population level.

From a public health perspective, tools designed to characterize temporal risk patterns and individual pain fluctuations represent promising avenues for personalized care. The Chronorisk framework, originally developed for cluster headache^42^, illustrates how temporal profiling may support individualized monitoring and counseling in trigeminal pain conditions that share overlapping biological and circadian features^43–45^. Such approaches could help to identify weather-sensitive phenotypes and refine patient-specific management strategies.

Long-term climate–health surveillance remains relevant. Although the climatic effects observed in this study were modest, progressive environmental changes and increasing frequency of extreme weather events may alter the epidemiology and healthcare burden of chronic pain conditions over time^4,9,12^. Integrating meteorological data with primary care surveillance systems, digital pain diaries, or wearable technologies could enhance the detection of individualized weather–pain relationships and support anticipatory healthcare planning^9,34,35^. Given the substantial societal burden of headache and neuropathic pain disorders^7,40^, even small improvements in symptom anticipation or healthcare utilization efficiency could translate into meaningful gains in the quality of life and resource allocation.

### Limitations and future directions

Several limitations of this study should be acknowledged when interpreting these findings. First, the use of routinely collected primary care administrative records is an inherent constraint. These records capture only the moment of formal contact between the patient and the healthcare system, and do not provide information on the latency between symptom onset and consultation. This delay, shaped by factors such as individual pain tolerance, self-medication, access to care, or initial misattribution of symptoms to dental or other conditions, means that consultation frequency reflects healthcare demand rather than the true temporal incidence of trigeminal neuralgia or facial neuropathic pain. As a result, the early or milder stages of the disease may be underrepresented.

A second important limitation relates to the COVID-19 pandemic period, which coincided with profound disruptions in healthcare delivery and patient behavior. Because primary care records reflect healthcare utilization rather than symptom onset, consultation patterns during and immediately after the pandemic may have been influenced by delayed care seeking, reduced service availability, or reprioritization of healthcare resources. Although this interval is visually indicated in Fig. 1 to provide a temporal context, the present study was not designed to isolate or quantify the causal impact of the pandemic. Therefore, the temporal changes observed during this period should be interpreted with caution.

Third, the relatively low incidence and sparse temporal distribution of trigeminal neuralgia consultations represent methodological challenges for conventional time-series approaches. This limitation motivated the transition from classical forecasting models to piecewise additive models, which allows individual consultation events to be analyzed as time-to-event data rather than aggregated counts. Although this approach improves analytical sensitivity in low-density datasets^25,26^, limited statistical power may still constrain the detection of modest associations, particularly for demographic covariates.

In this context, age and sex did not emerge as independent predictors in the multivariate model. This finding should be interpreted cautiously because the combination of a low number of cases and simultaneous adjustment for temporal and environmental covariates may attenuate detectable demographic effects. Importantly, the absence of independent associations at the population level does not preclude age or sex as baseline risk modifiers at the individual level.

Additional limitations include the use of meteorological data from a single AEMET station, which restricts spatial granularity and may not capture the local microclimatic variability across the study area. Moreover, other environmental exposures, such as air pollution, short-term humidity variability, or ambient noise, were not included, despite evidence suggesting that these factors may interact with pain perception and autonomic regulation^46,40^. The lack of patient-reported outcomes, including pain intensity, attack duration, trigger perception, or psychosocial context, further limits the ability to explore individual-level meteorosensitivity.

Finally, the inclusion of facial neuropathy cases coded under ICPC-2 N89.02 introduces a degree of etiological heterogeneity. Similarly, the use of administrative ICPC-2 codes does not allow reliable differentiation between classical and secondary trigeminal neuralgia or precise identification of specific etiologies underlying facial neuropathic pain at the time of primary care consultation, which is beyond the scope of the present analysis. Moreover, the structure of the administrative dataset did not allow reliable identification of whether individual patients were coded exclusively as trigeminal neuralgia or facial neuropathy or received both labels across different consultations. However, this choice also enhances external validity by reflecting real-world primary care diagnostic practices, where trigeminal neuralgia and related facial neuropathic pain conditions are frequently managed together. Accordingly, the findings should be interpreted as applicable to trigeminally mediated facial pain presentations in primary care rather than exclusively to classical trigeminal neuralgia.

Future research should aim to overcome these limitations by incorporating multicenter datasets with broader geographical coverage, higher case density, and finer spatial resolution for meteorological measurements. Integrating clinical records with neuroimaging, electrophysiological data, and psychosocial assessments may help to identify specific trigeminal pain phenotypes that are more susceptible to environmental modulation. Prospective designs that combine bioclimatic modeling with machine learning approaches could further enhance the predictive value of meteorological information for chronic facial pain management.

In addition, the use of ecological momentary assessment methodologies would allow real-time capture of pain dynamics, contextual exposures, and short-term climatic variability, providing more granular insight into potential weather–pain relationships that may not be detectable in administrative healthcare datasets^47^.

## Conclusions

This 14-year population-based time-series analysis provides novel evidence of the relationship between climatic variability and healthcare utilization for trigeminal neuralgia and facial neuropathic pain in primary care. Using a piecewise additive modeling approach tailored to low event density and non-linear temporal dynamics, specific climatic factors, namely hours of sunlight and low ambient temperature, were associated with an increased risk of seeking medical attention.

These findings suggest that climatic variables may function as contextual modulators of symptom perception and consultation behavior, rather than as primary drivers of disease incidence. In particular, a higher consultation risk at lower temperatures and during periods of increased sunlight exposure likely reflects a complex interaction between environmental conditions, individual sensitivity, behavioral factors, and access to care.

Importantly, intrinsic neuropathological mechanisms remain central to the pathophysiology of trigeminal neuralgia and related facial neuropathic pain syndrome. Environmental influences appear to exert selective, non-linear, and modest effects at the population level, consistent with the heterogeneous meteorosensitivity reported in chronic pain research.

From clinical and public health perspectives, these results support the integration of biometeorological awareness into individualized patient counseling, particularly for patients reporting reproducible weather-related symptom exacerbations. Future multicenter and prospective studies combining high-resolution meteorological data with patient-reported outcomes, neurophysiological markers, and ecological momentary assessments may help identify weather-sensitive phenotypes and refine personalized management strategies for trigeminal pain conditions.

## Supplementary Information

Below is the link to the electronic supplementary material.

Supplementary Material 1

## Data Availability

The data presented in this study are available upon request from the corresponding authors.
