# Supplementary material for: Impact of climatic factors on trigeminal neuralgia and facial neuropathy in primary care during a fourteen-year time-series study
Source: Sci Rep. 2026 Apr 22;16:18619. doi: 10.1038/s41598-026-49760-0 (PMC13270028; doi:10.1038/s41598-026-49760-0)
Supplement: Supplementary file 1 — Supplementary Material 1 [file 41598_2026_49760_MOESM1_ESM.docx]

**Supplementary material**

**Supplementary material. Table 1:**Variable selection and smoothing parameter diagnostics for the initial GAM model.

| **Variable** | **Decision** | **EDF** |
| --- | --- | --- |
| Time trend | Non-linear (Spline) | 4.343 |
| Age | Noise (Exclude) | 0.437 |
| Average temperature (degrees Celsius) | Non-linear (Spline) | 1.804 |
| Average rainfall (l/m²) | Noise (Exclude) | <0.001 |
| Average wind speed (mtrs/sec) | Noise (Exclude) | <0.001 |
| Wind gusts (mtrs/sec) | Linear (Parametric) | 0.798 |
| Sunshine hours | Linear (Parametric) | 0.815 |
| Diurnal temperature range (degrees Celsius) | Noise (Exclude) | <0.001 |
| Average barometric pressure (hPa) | Noise (Exclude) | <0.001 |
| Wind direction (cosine) | Linear (Parametric) | 0.76 |
| Wind direction (sine) | Noise (Exclude) | <0.001 |

EDF: Effective degrees of freedom.

This table summarizes the decision process for the inclusion or exclusion of candidate predictors based on the effective degrees of freedom (EDF) and penalization. EDF values close to zero indicate exclusion due to noise, whereas higher values reflect non-linear relationships modeled using splines.

**Supplementary material. Table 2:** Final model evaluation.

| **Smoothed terms** | | |
| --- | --- | --- |
|  | **Concurvity** | **K-index (p value)** |
| Average temperature (degrees Celsius) | 0.339 | 0.71 |
| Time trend | 0.032 | 0.657 |
| **Linear terms** | | |
|  | **VIF** |  |
| Sunshine hours | 0.008 |  |
| Wind gusts (mtrs/sec) | 0.005 |  |
| Gender (Male) | 0.053 |  |
| **Global diagnostics** | | |
|  | **p value** |  |
| Dunn-Smyth test | <0.001 |  |
| Box-Ljung test | 0.909 |  |

VIF: Variance inflation factor.

Statistically significant p-values and values outside the established cutoff ranges are highlighted in red.

Concurvity indices and K-index values were reported for smoothed terms to assess the non-linear dependency and adequacy of the basis dimension. Variance inflation factors (VIF) are shown for linear terms to evaluate multicollinearity. Global model diagnostics include Dunn–Smyth and Ljung–Box tests to assess the residual distribution and autocorrelation, respectively. Values outside of the predefined acceptable range are highlighted in red.
